# A Cross-Sectional Study of Antenatal Care Attendance among Pregnant Women in Western Jamaica

**DOI:** 10.4172/2376-127x.1000341

**Published:** 2017-07-31

**Authors:** Ebony Townsend Respress, Pauline E. Jolly, Chinye Osia, Nichole Dawson Williams, Swati Sakhuja, Suzanne E Judd, Maung Aung, April P Carson

**Affiliations:** 1 Department of Epidemiology, University of Alabama at Birmingham, USA; 2 Department of Biostatistics, University of Alabama at Birmingham, USA; 3 Epidemiology and Research Unit, Western Regional Health Authority, Ministry of Health, Jamaica

**Keywords:** Antenatal care services, Antenatal care knowledge, Pregnant women

## Abstract

**Background:**

Pregnancy complications are preventable with appropriate antenatal care (ANC). However, ANC attendance recommendations vary.

**Objective:**

This study investigated ANC practices and predictors of ANC visits among pregnant women in western Jamaica during 2010.

**Methods:**

A cross-sectional study was conducted among 356 pregnant women. ANC visits were categorized as not meeting recommendations (<4 ANC visits), meeting WHO and the Jamaican Ministry of Health recommendations of a minimum of 4 ANC visits (4–6 ANC visits) or meeting previously standard recommendation of ≥7 visits. Differences in demographic factors, health status, ANC services received and ANC knowledge by ANC attendance were assessed and a multinomial forward-selection stepwise logistic regression model was used to identify predictors of ANC attendance.

**Results:**

Most women had an adequate number of ANC visits with 53.4% attending ≥ 7 ANC visits and 27.2% attending 4–6 visits. Despite this, 19.4% of the women had inadequate ANC care and a large portion did not receive key ANC services such as folic acid supplementation (48%), information on breastfeeding (32%) and nutrition (13%). Employment status, number of live births, distance from clinic, history of diabetes or hypertension, possession of ANC card at delivery, receiving iron supplementation and HIV counseling and testing and antenatal care knowledge were predictors of ANC visits.

**Conclusion:**

Although most women met the WHO or Jamaican ANC recommendations, many women still did not receive key ANC services. Further investigation of ANC practices and a standardized ANC curriculum may improve provision of adequate ANC services.

## Introduction

A significant health disparity exists between developing and developed countries for maternal and infant health outcomes [1]. Poverty, lack of infrastructure and inadequate healthcare contribute to developing countries bearing the greatest maternal and infant mortality burden, with 99% of all maternal and newborn deaths occurring in low or middle-income countries [2,3]. Pregnant women in developing countries have an increased risk for pregnancy-related complications and death and the infants of these mothers have an increased risk for complications during birth and shortly after delivery [4,5]. However, many of the complications are preventable with appropriate antenatal care (ANC) [6–10].

The World Health Organization’s (WHO) recommendation of at least four ANC visits, spaced across regular intervals, and with a skilled attendant has been shown to improve health outcomes for both expectant mothers and infants [11,12]. The WHO contends that the first antenatal care visit should occur with a skilled health attendant and as early as possible in the first trimester [13]. For optimal health outcomes, comprehensive ANC should contain each of the following components, (1) identification of pre-existing conditions such as anemia, HIV or hypertension (2) early detection of complications arising during pregnancy, such as gestational diabetes or preeclampsia (3) health promotion and disease prevention, including vaccines, nutrition counseling and micronutrient supplements and (4) birth preparedness and complication planning, including breastfeeding counseling and antiretroviral therapy for HIV positive women [14]. Nearly half of all pregnancies in developing countries are not monitored by skilled healthcare professionals [15]. Moreover, women who do receive some ANC generally have irregular visits, large spacing between visits and poor communication with healthcare providers throughout pregnancy [16]. While ANC policies have improved globally, many low-income pregnant women do not receive the recommended number of ANC visits and often initiate and attend visits late in their pregnancies [17,18].

In Jamaica, 98% of expectant mothers have at least one antenatal visit during their pregnancy and 87.1% have at least four ANC visits [19]. Despite most women adhering to WHO and Jamaican Ministry of Health ANC recommendations, women in rural areas of Jamaica have the poorest ANC attendance [20]. In an effort to combat the maternal and infant morbidity and mortality, the Jamaican Ministry of Health, although following the WHO recommendation of a minimum of four antenatal visits per pregnancy, encouraged seven antenatal visits and provided ANC booklets to record important information about the mother and infant’s health to facilitate better ANC coordination [21,22].

Few studies have investigated the current ANC practices of pregnant women in western Jamaica. Thus, the purpose of this study was to investigate ANC practices among pregnant women, in this region of Jamaica, in 2010, according to WHO and Jamaican Ministry of Health’s recommendations.

## Methods

### Study design and participants

A cross-sectional study was conducted among pregnant women who presented to the delivery suites at four hospitals within the Western Regional Health Authority of Jamaica (Cornwall Regional Hospital, Savanna La Mar Hospital, Falmouth Hospital and Noel Holmes Hospital) between May and September 2010. During this period, 410 pregnant women consented to participate in this study and data were obtained from interviewer-administered questionnaires and the mothers’ clinic charts. Women with incomplete ANC data (N=54) were excluded from this study resulting in 356 women for this analysis. This study was approved by the Institutional Review Board (IRB) of the University of Alabama at Birmingham, the Advisory Panel of Ethics and Medico-Legal Affair in the Jamaican Ministry of Health and the Western Regional Health Authority.

### Demographics and medical history

Age, marital status, education, occupation and income were self-reported. Age was categorized as 19–25, 26–32, or ≥ 33 years of age. Marital status was categorized as single (including those divorced and widowed), married, or cohabitating. Highest level of education was categorized as ≤ Primary School Education, Secondary Education, Technical/Vocational and College/University. Employed women were those working for pay or self-employed at the time of interview. Unemployed included women who were housewives, students, or retired. Weekly household income was categorized as ≤ $J2599 (~$21.70USD/week), between $J2600 and $J4999 (~$21.71 to $41.73USD/week) and ≥ $J5000 ($41.74USD/week).

Interview data and ANC records booklet were used to assess medical history. Women were asked whether this was their first pregnancy, whether they were utilizing contraception at the time they became pregnant and the number of live children to whom they had given birth. In addition, self-reported history of hypertension or diabetes was recorded.

### Antenatal care

The number of ANC visits was self-reported and was used to categorize the women’s ANC attendance as not meeting recommendations (<4 visits), meeting WHO and the Jamaican Ministry of Health recommendation (4–6 visits) or meeting previously standard recommendation of ≥7 visits. Additionally, whether the women did or did not bring their ANC card to delivery was recorded.

The type of ANC clinic that the women attended was categorized as public only or a combination of public and private facilities. The mothers’ distance from their ANC clinic was categorized as 0–5 miles, 6–10 miles, or more than 10 miles and women were asked whether they had their own transportation available.

Receipt of ANC services was self-reported and defined by whether the women received iron supplementation, folic acid supplementation, information on breastfeeding, information on nutrition during pregnancy and voluntary counseling and testing for HIV. Satisfaction with ANC services was assessed by asking the women if all their questions were answered during their ANC visits and whether they were satisfied with the quality of service at their visits.

The participants’ ANC knowledge was assessed by asking whether they learned new information during their ANC visits, by determining whether they were able to correctly identify the trimester when the first ANC visit should occur and by correctly identifying the stage of pregnancy when fetal deformities were most likely to occur.

### Statistical analysis

Univariate analysis was conducted to summarize the demographic characteristics, ANC attendance, possession of ANC card at delivery, health status, ANC services received and ANC knowledge. To determine if there were significant differences in ANC attendance among the women, frequencies and percentages were calculated by number of ANC visits attended, categorized as not meeting recommendations (<4 ANC visits), meeting the WHO and the Jamaican Ministry of Health ANC recommendation (4–6 ANC visits) or meeting previously standard recommendation of ≥7 visits. Chi-square or Fisher’s exact tests were performed for categorical variables to obtain p-values.

Independent associations of demographic factors, possession of ANC card, health status, services received and knowledge of number of ANC attendance were assessed using a multinomial forward stepwise logistic regression model. Variables which met the P<=15% significance level were included in the model. Those women who met the standard ANC recommendation of ≥ 7 visits were used as the referent group for analysis and compared to the women with inadequate ANC visits WHO and Jamaican ANC recommendation. Odds ratios (ORs) and their corresponding 95% confidence intervals (CIs) were computed. Ail analyses were two tailed at an alpha level of p<0.05. Data was analyzed using SAS software, Version 9.4 (SAS Institute, Cary NC).

## Results

### Study population

Most of the women within this sample received an adequate number of ANC visits. The majority of the women, 80.6% met Jamaican or WHO ANC recommendations of at least 4 ANC visits. Despite this, 19.4% of the women had inadequate ANC care with less than the recommended four visits. Almost half (49.7%) of the participants were aged 19–25 and 23.0% were age 33 or older. Most of the women identified as single, divorced or widowed (47.5%). Most of the participants (57.3%) had at least some secondary education, while 9.3% had a primary school education or less. More than half (57.9%) of the women were employed and 57.0% reported a weekly household income of ≥ $J5000 ($41.74USD/week). The majority of the participants (81.2%) came from the Cornwall Regional Hospital and most of the women attended ANC al public clinics (79.8%). Approximately half of the women had ≤ 5 miles to travel to the ANC clinic in contrast to 27.2% who had more than ten miles lo travel. More than half (61.5%) of the women did not have personal transportation to attend the ANC clinic (Table 1).

Iron supplementation (91.0%), folic acid supplementation (51.7%) and voluntary counseling and testing for HIV (94.1%) were common. In addition, the majority of women reported receiving information on breastfeeding (67.7%) and information on nutrition (87.1%). Most of the women (84.8%) reported that they had all their questions answered during their ANC visits and were satisfied with the quality of service (74.2%). However, more than 1 in 4 of the women (27.5%) indicated that they did not learn any new information during their ANC visits. Most of the women (83.2%) were able to correctly identify that a pregnant woman should have her first ANC visit during the first trimester. However, 61.8% of the women were unable to correctly identify that first trimester was the stage of pregnancy with the greatest likelihood of birth defects occurring (Table 2).

### Predictors of antenatal care attendance

The predictors retained in the model were employment status, number of live births, mother’s distance from the clinic, history of diabetes or hypertension, possession of ANC card at delivery, receipt of iron supplementation, voluntary counseling and testing for HIV, correct identification of the trimester with the greatest likelihood for deformity, and correct identification of when the first ANC exam should occur. Women who were unemployed were more likely to have <4 ANC visits (OR=1.9, 95% CI=0.9–3.5) when compared to women who were employed but the association was not significant. Women with more than ten miles to travel to an ANC clinic were more likely to have <4 ANC visits (OR=2.2, 95% CI=1.1–4.5). Women with a history of diabetes or hypertension were less likely to attend <4 ANC visits (OR=0.4, 95% CI=0.2–1.1) or 4–6 visits (011=0.6, 95% CI=0.3–1.2), when compared to women without these chronic illnesses but the association was not significant. Women who did not bring their ANC card to delivery were more likely to have <4 ANC visits (OR=3.8, 95% CI=1.9–7.3) when compared with women who did bring their ANC card to delivery, whereas there was no association for bringing ANC card to delivery for women with 4–6 ANC visits (OR=1.0, 95% 0=0.6–1.9).

Women who did not receive iron supplementation were more likely to report having <4 ANC visits (OR=4.1, 95% CI= 1.5–11.3) when compared to women who did receive iron supplementation. Women who did not receive voluntary counseling and testing for HIV were more likely to report having <4 ANC visits (OR=3.5, 95% 0=1.1–12.2) when compared to women who did not receive HIV testing and counseling.

Women who were unable to identify the stage of pregnancy with the greatest likelihood of deformity for the newborn were more likely to have <4 ANC visits (OR=2.6, CI=1.3–5.1) or 4–6 visits (OR=2.1, 95% CI=1.2–3.6) than women who were able to make a correct identification. Women who were unable to identify when the first ANC examination should occur were more likely to have <4 ANC visits (OR=3.2, 0=1.4–7.1) (Table 3).

## Discussion

Findings from this study indicate that while the majority of Western Jamaican women have at least four ANC visits, there is still a disparity within the western region of the country. Recent national data indicate that 87.1% of women receive at least four ANC visits in Jamaica. When comparing this proportion to the prevalence within this sample of pregnant women, we find a slightly larger proportion (19.4%) of women had less than 4 ANC visits. Specifically, there is a relatively large group of women who are receiving no or inadequate ANC throughout their pregnancies in Western Jamaica [23].

Given this disparity among women in the region, it is important to study the factors that may impact ANC attendance within this group of women. Findings from the present study suggest that socioeconomic factors, such as unemployment, can negatively impact the number of ANC visits women attend. Economic pressures may decrease the women’s ability to attend the recommended number of ANC visits [24]. In addition, though education was weakly associated with number of ANC visits in this study, it may play a role in whether the women understand the importance of attending regular ANC visits, as studies have shown that education is positively associated with health services utilization [25].

Pregnant women with a history of diabetes and hypertension were more likely to meet WHO or Jamaica’s ANC recommendations in this study. This is important to note because women with diabetes or hypertension are at particularly high risk for pregnancy and birth complications, such as preeclampsia, placental abruption and premature delivery [26,27].

Iron supplementation, receiving counseling and testing for HIV and having all questions answered during ANC visits were associated with more ANC visits. This was anticipated as more ANC visits would better provide an opportunity for women to receive these important health services. However, a large portion of women (48.3%) did not receive folic acid supplementation, which is necessary to prevent serious birth defects of the brain and spinal cord [28]. Moreover, a sizeable proportion of women did not receive information on nutrition during pregnancy (12.9%) despite differences in nutritional needs for pregnant women or breastfeeding (32.3%) [29]. It is also important to note that a significant portion of those women who did not receive information on breastfeeding were first lime mothers (almost 40%). Breastfeeding has been demonstrated lo decrease risk for health complications for both the mother and the baby. For this reason, new mothers, especially, should be encouraged and provided information on the benefits of breastfeeding [30].

The women’s knowledge of ANC factors was associated with the more ANC visits they had attended. However, 24.6% of the women who met WHO or Jamaican ANC recommendations could not identify when the first ANC visit should occur. Awareness of the stage of pregnancy when newborn deformity is most likely was also poor with 20.6% of the women who met WHO or Jamaican ANC recommendations unable to correctly identify this critical period in pregnancy. Given these findings, the women’s inability to correctly identify basic benefits of ANC attendance is indicative of an ANC knowledge gap. It may be beneficial to increase ANC education within Jamaican communities to ensure that all women understand the importance and benefits of ANC attendance.

Differences in folic acid supplementation, voluntary HIV testing and counseling and ANC knowledge were apparent for women attending ≥ 7 visits compared with 4–6 visits. As many other variables were similar for those attending ≥ 7 visits or 4–6 visits, this begs the question whether the additional ANC visits, beyond four visits, are necessary for women without complicated pregnancies or comorbidities. These additional ANC visits may create an undue burden on pregnant women in this region. In addition, the additional visits may not be effectively making use of resources devoted to maternal and infant health [16]. Future studies should investigate associations with maternal and child outcomes to determine the benefit of additional ANC visits in addition to exploring whether the content of ANC visits vary across the western region.

Findings further indicated that a large portion of the pregnant women did not bring their ANC cards to the delivery suites, as women with more ANC visits were less likely to bring their cards to delivery. This is an important factor as providing an accurate and complete antenatal care record to the healthcare provider is essential to ensuring a healthy delivery. However, in developing countries, like Jamaica, medical record collaboration among providers may be difficult. Since ANC is often performed by varying providers at different locations, prenatal records are often missing or incomplete at delivery. It is incumbent on the women that they maintain and take their ANC card to their clinic visits and delivery to ensure proper service. Not having these records available can leave the mother and child vulnerable to a myriad of health complications [31].

While these findings reveal important predictors of ANC attendance in western Jamaica, there are several potential limitations to consider when interpreting these results. The cross-sectional study design does not allow for causal inference and there was limited power given the small sample size. Participants in this study were recruited from medical facilities, so these findings may not be generalizable to all women in western region who do not access these services. The data were self-reported and may be subject to social desirability bias [32]. It is possible that participants answered survey questions in a manner consistent with what is deemed socially or morally acceptable instead of reporting actual beliefs or behaviors. Additionally, there were no data available about during which trimester the ANC visits occurred, limited information on comorbidities and no information about the postnatal status of the mother or infant. Finally, as per data from the World Bank, only 2.3% of women in Jamaica did not receive prenatal care, therefore, not including women who did not attend ANC facilities may not have had much impact on the results of this study [33].

In summary, this study demonstrated that women in Western Jamaica are often not receiving standard ANC services, although the majority (80%) is meeting WHO and Jamaican ANC visit recommendations. Our study showed that key ANC services such as folic acid supplementation, nutrition information and information on breastfeeding were not received by many women regardless of number of ANC visits attended. Therefore, ANC providers in Jamaica will need to ensure that all of these aspects are included in the care of every pregnant woman to improve upon the maternal and infant mortality and morbidity within the western region.

## Conclusion

Although most women met the WHO or Jamaican ANC recommendations, many women still did not receive key ANC services. Further investigation of ANC practices and a standardized ANC curriculum may improve provision of adequate ANC services.

## Figures and Tables

**Table 1:** Participant characteristics by antenatal care attendance in Western Jamaica, 2010.

	Participants	<4 Antenatal Care Visits	4–6 Antenatal Care Visits	≥ 7 Antenatal Care Visits	P-Value
	(N=356)	(N=69)	(N=97)	(N=190)	
					
	N (%)	N (%)	N(%)	N (%)	
**Age**					
19–25 years	177 (49.7)	39 (56.5)	51 (52.6)	87 (46.8)	0.5957
26–32 years	97 (27.3)	16 (23.2)	25 (25.8)	56 (29.5)	
≥ 33 years	82 (23.0)	14 (20.3)	21 (21.6)	47 (24.7)	
**Educational level**					
≤ Primary School	33 (9.3)	10 (14.5)	10(10.3)	13 (6.8)	
Secondary	204 (57.3)	41 (59.4)	47 (48.4)	116 (61.1)	
Technical/Vocational	49 (13.7)	7 (10.1)	19 (19.6)	23 (12.1)	
College/University	70 (19.7)	11 (16.0)	21 (21.7)	38 (20.0)	
**Marital status**					
Single (single, divorced, widowed)	169 (47.5)	41 (59.4)	41 (42.3)	87 (45.6)	0.2445
Cohabitating	133 (37.4)	21 (30.4)	39 (40.2)	73 (38.4)	
Married	54 (15.1)	7 (10.1)	17 (14.5)	30 (15.8)	
**Employment status**					
Employed	206 (67.9)	33 (47.8)	52 (53.6)	121 (63.7)	0.0448*
Unemployed	150 (42.1)	36 (52.2)	45 (46.4)	69 (36.3)	
**Household income per week**					
≤ J$2599	104 (29.2)	27 (39.1)	32 (33.0)	45 (23.7)	0.0889
Between JS2600-J$4999	49 (13.8)	11 (16.0))	12 (12.4)	26 (13.7)	
≥ J$5OOO	203 (57.0)	31 (44.9)	53 (54.6)	119 (62.6)	
**Type of clinic**					
Public	284 (79.8)	59 (85.5)	80 (82.5)	145 (76.3)	01.967
Private or Combination	72 (20.2)	10 (14.5)	17 (17.5)	45 (23.7)	
**Distance from clinic**					
0–5 miles	170 (47.8)	31 (45.0)	39 (40.2)	100 (52.6j	0.2270
6–10 miles	89 (25.0)	15 (21.7)	29 (29.9)	45 (23.7)	
10+ miles	97 (27.2)	23 (33.3)	29 (29.9)	45 (23.7)	
**Live births ≠ current pregnancy**					
None	167 (44.1)	36 (52.2)	42 (43.3)	79 (41.5)	0.2240
One child	80 (22.5)	10 (14.5)	24 (24.7)	46 (24.2)	
Two children	47 (13.2)	9 (13.0)	17 (17.5)	21 (11.1)	
Three or more children	72 (20.2)	14 (20.3)	14 (14.5)	44 (23.2)	
**First pregnancy**	148 (41.6)	33 (47.8)	40 (41.2)	75 (39.5)	0.4819
**Own transportation to clinic**	137 (38.5)	21 (30.4)	38 (39.2)	78 (41.0)	0.2956
**Using contraception at time of pregnancy**	221 (62.1)	43 (62.3)	54 (55.7)	124 (65.3)	0.2847
**History of diabetes or hypertension**	66 (18.5)	9 (13.0)	14 (14.4)	43 (22.6)	0.1018
**Brought ANC card to delivery**	253 (71.1)	36 (52.2)	75 (77.3)	142 (74.7)	0.0005*

* P-values for categorical variables are for differences in prevalence between numbers of antenatal care visits and were obtained from chi-square test

* Denotes statistically significant findings

**Table 2:** Antenatal care services and participant knowledge by number of antenatal care visits attended.

	Participants	<4 Antenatal Care Visits	4–6 Antenatal Cars Visits	≥ 7 Antenatal Care Visits	P-Value
	(N=356)	(N=69)	(N=97)	(N=190)	
	N (%)	N(%)	N(%)	N(%)	
**Antenatal Caro Services**					
Received iron supplementation	324 (91.0)	57 (82.6)	87 (89.7)	180 (94.7)	0.0092*
Received folic acid supplementation	184 (51.7)	29 (42.0)	50 (51.6)	105 (55.3)	0.1694
Received counselling and testing for HIV	335 (94.1)	61 (88.4)	90 (92.8)	184 (96.8)	0.0316*
Received information on breastfeeding	241 (67.7)	43 (62.3)	71 (73.2)	127 (66.8)	0.3139
Received information on nutrition	310 (87.1)	59 (85.5)	88 (90.7)	163 (85.8)	0.4547
Had all questions answered during visits	302 (84.8)	59 (85.51)	75 (77.3)	168 (88.4)	0.0455*
Satisfied with quality of ANC visits	264 (74.2)	53 (76.8)	69 (71.1)	142 (74.7)	0.6874
**Antenatal Care Knowledge**					
Learned new information during visits	258 (72.5)	52 (75.4)	70 (72.2)	136 (71.6)	0.8313
Able to Identify trimester with greatest likelihood of deformity	296 (B3.2)	52 (75.4)	77 (79.4)	167 (87.9)	0.0006*
Able to identify trimester when first ANC exam should occur	136 (38.2)	17 (24.6)	29 (29.9)	90 (47.4)	0.0299*

* P-values for categorical variables are for differences in prevalence between number of antenatal care visits and were obtained from chi-square test

* Denotes statistically significant

**Table 3:** Predictors for not meeting Jamaican ANC standards in forward-stepwise logistic regression model.

	<4 Antenatal Care Visits	4–6 Antenatal Care Visits	P-Value
**Participant Characteristics**			
**Employment status**			
Employed	**Reference**	**Reference**	
Unemployed	1.9 (0.9–3.5)	1.5 (0.9–2.6)	0.0966
**Number of live births ≠ current pregnancy**			
None	**Reference**	**Reference**	
One Child	0.4 (0.2–1.1)	1.1 (0.5–2.0)	
Two Children	1.4 (0.5–3.9)	1.9 (0.9–4.3)	0.1222
Three or more children	0.7 (0.3–1.5)	0.6 (0.3–1.3)	
**Distance from clinic**			
0–5 miles	**Reference**	**Reference**	
6–10 miles	0.9 (0.4–2.2)	1.6 (0.9–3.1)	
10+ miles	2.2 (1.1–4.5)	1.9 (0.9–3.5)	0.0789
**History of Diabetes or Hypertension**			
None	**Reference**	**Reference**	
Diabetic or Hypertensive	0.4 (0.2–1.1)	0.6 (0.3–1.2)	0.0969
**Brought ANC card to delivery**			
Yes	**Reference**	**Reference**	
No	3.8 (1.9–7.3)	1.0(0.6–1.9)	0.0001*
**Antenatal Care Services**			
**Received iron supplementation**			
Yes	**Reference**	**Reference**	
No	4.1 (1.5–11.3)	2.4 (0.9–6.3)	0.0238*
**Received counseling and testing for HIV**			
Yes	**Reference**	**Reference**	
No	3.5 (1.1–12.2)	2.4 (0.7–8.1)	0.1273
**Antenatal Care Knowledge**			
Able to identify trimester with greatest likelihood of deformity			
Yes	**Reference**	**Reference**	
No	2.6 (1.3–5.1)	2.1 (1.2–3.6)	0.0024*
Able to identify trimester when first ANC exam should occur			
Yes	**Reference**	**Reference**	
No	3.2(1.4–7.1)	1.9 (0.9–3.9)	0.0162*

* Values obtained from forward stepwise multinomial logistic regression. Denotes statistically significant
